# Risk assessment and prognostic analysis of patients with splenic infarction in emergency department: a multicenter retrospective study

**DOI:** 10.1038/s41598-021-00897-0

**Published:** 2021-11-02

**Authors:** Chieh-Ching Yen, Chih-Kai Wang, Shou-Yen Chen, Shi-Ying Gao, Hsiang-Yun Lo, Chip-Jin Ng, Chung-Hsien Chaou

**Affiliations:** 1 Department of Emergency Medicine, Chang Gung Memorial Hospital, Linkou Branch, Taoyuan, Taiwan, ROC; 2 College of Medicine, National Yang-Ming University, Taipei, Taiwan, ROC; 3 College of Medicine, Chang Gung University, Taoyuan, Taiwan, ROC; 4 Chang Gung Medical Education Research Center, Taoyuan, Taiwan, ROC; 5 Department of Emergency Medicine, Chang Gung Memorial Hospital, Linkou and Chang Gung University College of Medicine, Taoyuan, Taiwan, ROC

**Keywords:** Thromboembolism, Risk factors

## Abstract

Splenic infarction is a thromboembolic disease that is frequently missed in acute settings. Previous reviews were rarely presented from a clinical perspective. We aimed to evaluate the clinical characteristics, risk factors with diagnostic value, and prognostic factors using large cohort data and a matched case–control study method. A retrospective medical record review of six hospitals in Taiwan from January 1, 2005, to August 31, 2020, was conducted. All patients who underwent contrast CT with confirmed the diagnosis of splenic infarction were included. Their characteristics were presented and compared to a matched control group with similar presenting characteristics. Prognostic factors were also analyzed. A total of 130 cases were included, two-thirds of whom presented with abdominal pain. Atrial fibrillation was the most common associated predisposing condition, followed by hematologic disease. A higher proportion of tachycardia, positive qSOFA score, history of hypertension or atrial fibrillation, leukocytosis, and thrombocytopenia were found in splenic infarction patients compared to their counterparts. An underlying etiology of infective endocarditis was associated with a higher proportion of ICU admission. Splenic infarction patients often presented with left upper abdominal pain and tachycardia. A history of hypertension, atrial fibrillation, a laboratory result of leukocytosis or thrombocytopenia may provide a clue for clinicians to include splenic infarction in the differential list. Among the patients diagnosed with splenic infarction, those with an underlying etiology of infectious endocarditis may be prone to deterioration or ICU admission.

## Introduction

Splenic infarction is a frequently missed diagnosis in acute clinical settings. It is often under-diagnosed because it has a wide range of non-specific clinical presentations and underlies fresh-diagnosed illness^1^. Splenic infarction develops when an embolus or in situ thrombosis obstructs any of four to five splenic artery branches^2^. Cardioembolism and identifiable hypercoagulable states have been considered to be the cause of most splenic infarctions^3^. Although an incidence rate of 0.016% of admissions was documented in a previous study^1^, it was also reported that only 10% of patients diagnosed with splenic infarction were found antemortem^4^.

Splenic infarction is known to present with left upper quadrant (LUQ) abdominal pain, nausea, and vomiting^1,5,6^. Other possible complaints including left flank pain, left pleuritic chest pain, and fever with chills^7^. For laboratory findings, leukocytosis has been reported to be associated with single splenic infarction^8^. The fastest and preferred noninvasive radiologic tool for splenic infarction is an abdominal echography. The computed tomographic (CT) scans with contrast enhancement is another image of choice^9^. Due to escalating accessibility of computed tomographic(CT) scans^10–13^, splenic infarction is frequently an incidental finding and considered insignificant by emergency physicians. While it is often asymptomatic, splenic infarction may be an initial sign for other underlying serious conditions such as hematologic disease, malignancy, or cardioembolism^1,3,6–8^. Splenic infarction may further results in pseudocyst, abscess, hemorrhage, or rupture. Although these are rare and most patients recover without significant sequela, splenectomy may be needed when internal bleeding or sepsis occurs. Physicians should target the treatment at the source of the disease.

Previous studies are limited to autopsy or surgery-based cases, radiological image database, or clinical database with limited case numbers^1,3–8,14,15^. There were only five larger case series of splenic infarction patients who were diagnosed by CT. Brett et al. and Cox et al. presented 163 and 123 cases based on radiology databases^3,6^. Schattner et al. and Lawrence et al. recorded 32 and 28 cases with splenic infarction diagnosis before these patients discharged from the hospital^1,8^. Another study conducted by Antopolsky et al. included 48 cases based on an emergency department sample^7^. With limited comparisons from a clinical perspective, it is difficult to understand how patients with splenic infarction differ from others and who tend to deteriorate. This study aimed to evaluate the clinical characteristics, risk factors with diagnostic value, and prognostic factors using a large retrospective cohort data and a matched case–control study method.

## Materials and methods

### Study design and setting

This was a retrospective matched case–control cohort study utilizing regularly collected electronic medical records (EMR). The study sites were six hospitals in Taiwan which used the same EMR system, including two tertiary medical centers, three regional hospitals, and one district hospital. The capacity of the study sites combined was a total of over 9000 beds and an annual ED visit of 500,000 patients. All adult patients who met the inclusion criteria in the study sites from January 1, 2005, to August 31, 2020, were enrolled for analysis. This study was carried out in accordance with the STROBE guideline. This study was approved by the Chang Gung Medical Foundation Institutional Review Board (IRB no. 202001836B0) and was qualified for a waiver of informed consent. All methods were performed in accordance with the relevant guidelines and regulations.

### Patient selection and data collection

All adult patients with an ED or admission discharge diagnosis of splenic infarction (ICD-9: 289.59, ICD-10: D735) during the study period were enrolled in the study as cases. The electronic medical records (EMRs) of all enrolled patients were reviewed by two emergency physicians (CCY and CKW) for the accuracy of the diagnosis and relevant medical information. Patients under 18 at discharge, incomplete medical records, or the protected population due to the original IRB approval were excluded. For the cases, charts were reviewed, and the variables collected include age, gender, ED triage level according to a 5-level Taiwan Triage and Acuity Scale (TTAS) system. Their initial symptoms and signs, laboratory results, images, length of hospital stay, and underlying diseases were also recorded. Patient outcome variables collected include surgery, ICU admission, hospital length of stay, and mortality.

For over half of the patients with splenic infarction complained of left upper quadrant (LUQ) pain on arriving at the ED, we used a chief complaint-based approach to identify potential control patients. All patients who visited the ED with a chief complaint of LUQ pain and who had their blood samples taken during the study period were eligible for the control group. From this dataset, matched patients were selected at a 1:3 ratio using the Greedy algorithm^16^. Variables included in the matching process were age (± 5 years), sex, and triage acuity level using a 5-level Taiwan Triage and Acuity Scale (TTAS) system. The EMRs of the control group were also reviewed by authors, including the abdominal CT reports if the exams were being arranged. The relevant covariates of the control patients were then retrieved from the EMR system for subsequent analysis.

### Statistical analysis

For descriptive results, the continuous variables were presented using mean (SD), and the categorical variables were presented using count (%). For the comparison between independent groups, independent T-tests and chi-square tests were used to compare continuous and categorical variables, respectively. We compared the LUQ patients from the case and the control groups using a Generalized Estimating Equation (GEE) model with a logit link to account for the matched design^17^. For the analysis of the prognostic factor among the cases, univariate and multivariate logistic regression models were built using ICU admission or mortality as the binary endpoint. Several continuous explanatory variables were categorized into binary outcomes in the comparison or modeling process for better clinical interpretation. The cut-off values of each variable are the upper or lower limit of the normal range in the study site. A p-value of less than 0.05 was considered statistically significant. All statistical analysis was done using sas version 9.4^18^.

### Ethics approval and consent to participate

The study was approved by the Chang Gung Medical Foundation Institutional Review Board (IRB no. 202001836B0).

### Consent for publication

This study was qualified for a waiver of informed consent.

## Results

### Descriptive results

During the study period, 281 patients were discharged with the diagnosis of ICD-9: other diseases of spleen (289.59) or ICD-10: infarction of spleen (D735). After the chart review by two emergency physicians, 141 patients were excluded due to a lack of a definite diagnosis of splenic infarction. It was because that no specific coding in ICD-9 for splenic infarction and the coding: other diseases of spleen (289.59) included the splenic disorders other than splenic infarction. Ten patients were excluded owing to incomplete medical records. Finally, 130 patients were eligible to be included in the case group. All patients were admitted to the hospital. Among them, 18 were admitted to the ICU during their admission, and nine patients died with an overall mortality rate of 6.92% (Table 1). The patients' average age was 61.5 (SD = 16.1) years, and 58.5% were male. All patients were of Asian ethnicity. The proportions of the cases stratified using a TTAS 5-level triage from level 1 to level 4 was 3.9%, 30%, 62.3%, and 3.9%. Over two-thirds (71.5%) of the patients presented to the emergency department with abdominal pain, in which 72% localized to the left upper quadrant, followed by the epigastric region being the second most frequent location. The associated GI symptoms, including anorexia or nausea/vomiting, were present in 13% and 20% of the patients. Abnormal vital signs, including fever (BT > 38 °C), tachypnea (RR > 20 bpm), and tachycardia (HR > 100 bpm), were reported in 10.8%, 10.8%, 34.6% of the patients. Thirty patients (23%) had a positive qSOFA score (Table 2).

**Table 1:** Descriptive results of the patients with splenic infarction.

	Count (%)		Count (%)
Age^a^	61.5 (16.1)	**Past medical history**	
Male	76 (58.5)	Atrial fibrillation	32 (24.6)
**Initial vital sign**		Hematologic disease	21 (16.2)
Age > 65-year-old	59 (45.4)	Non-hematologic malignancy	13 (10.0)
Fever > 38 °C	14 (10.8)	Thromboembolism history	6 (4.62)
Hypotension	3 (2.31)	Liver cirrhosis	15 (11.5)
Tachycardia	45 (34.6)	Hypertension	60 (46.2)
Tachypnea	14 (10.8)	Diabetes mellitus	30 (23.1)
GCS < 14	4 (3.08)	Chronic kidney disease	16 (12.3)
**Triage**		Ischemic heart disease	18 (13.9)
1	5 (3.85)	Previous stroke	11 (8.46)
2	39 (30.0)	Congestive heart failure	16 (12.3)
3	81 (62.3)	Operation history	9 (6.92)
4	5 (3.85)	Immobile history	5 (3.85)
5	0 (0.00)	Healthy^b^	19 (14.6)
ICU admission	18 (13.8)		
Death	9 (6.92)		
Length of stay	14.2 (12.6)		

^a^Presented as mean (SD).

^b^If the patients did not have any medical history or comorbidities and did not take any medication, they will be classified to “healthy”.

**Table 2:** Clinical features of the 130 splenic infarction patients.

	Count (%)		Count (%)
**Initial presentation**		**Laboratory exam**	
Abdominal pain (regardless of location)	93 (71.5)	WBC > 10,000	77 (64.7)
LUQ pain	67 (51.5)	Hemoglobin < 8	8 (6.72)
Epigastric pain	12 (9.20)	Platelet > 450,000	10 (7.69)
Abdominal pain at other location	20 (15.4)	Platelet < 100,000	30 (23.1)
Left flank pain	6 (4.60)	Creatinine > 2	5 (4.55)
Back pain	20 (15.4)	Estimated GFR < 60	32 (30.8)
Dyspnea	12 (9.23)	LDH > 250	9 (6.92)
Cold sweating	8 (6.15)	AST > 120	5 (3.85)
Anorexia	17 (13.1)	Evidence of bacteremia	19 (15.5)
Nausea/vomiting	26 (20.0)	**qSOFA**	
**Diagnostic image**		0	100 (76.9)
Abdominal plain film	43 (33.1)	1	28 (21.5)
Abdominal echo	13 (10.0)	2	1 (0.77)
CT	130 (100)	3	1 (0.77)

Past medical histories were found in 95 of 130 patients (73.1%) (Table 2). The most common predisposing condition was hypertension (60/130,46.2%), followed by atrial fibrillation (28/130, 21.5%), diabetes (30/130,23.1%), and hematologic disease (22/130, 16.9%). Among the patients with the underlying hematologic disease, six had leukemia, five had lymphoma, six had polycythemia vera, one had essential thrombocythemia, and four had primary myelofibrosis. In the laboratory exams, 77 of 130 patients (64.7%) had elevated white blood cells (> 10,000 μL). Thrombocytosis (> 450 × ${10}^{9}$/L) and thrombopenia (< 100 × ${10}^{9}$/L) were found 10 (7.69%) and 30 (23.08%) of 130 patients, respectively. All patients received CT with contrast for the diagnosis of splenic infarction. A typical CT pattern may reveal a peripheral wedge-shaped hypodense lesion of the spleen. A plain abdominal film was performed in 43 patients (33%) and abdominal sonography in ten patients (10%).

### LUQ pain patients with and without splenic infarction

A total of 377 patients were sampled to the control group after matching age, sex, and triage acuity level among adult LUQ patients. Among them, 107 had abdominal CT arranged during their hospital stay, and non had splenic infarction documented. A comparison between these two groups is presented in Table 3. The splenic infarction patients showed a significant higher proportion with fever (10.2% vs. 2.65%, p < 0.01), tachycardia (34.4% vs. 17.0%, p < 0.001), and a positive qSOFA score (21.9% vs. 12.2%, p < 0.05). The case group showed a significantly different pattern in the past medical history compared to the control group. Patient with splenic infarction was more likely to have hypertension (46.1% vs. 10.1%, p < 0.001) and atrial fibrillation (23.4% vs. 8.22%, p < 0.001), and less likely to have diabetes mellitus (p < 0.001), chronic kidney disease (p < 0.001), chronic obstructive pulmonary disease (p < 0.001), chronic liver disease (p < 0.001), malignancy of any kind (p < 0.001), or congestive heart failure (p < 0.001) than the background population. The case group also revealed a higher rate of having leukocytosis (65.0% vs. 30.2%, p < 0.001) and thrombocytopenia (23.9% vs. 6.97%, p < 0.001). In prognosis, patients with splenic infarction showed a longer total hospital length of stay (p < 0.001), higher ICU admission rate (p < 0.001), and higher mortality rate (p < 0.001) than their counterparts.

**Table 3:** Comparison of patients who were and were not splenic infarction cases. The two groups were matched by age, gender, and ED triage levels.

	Splenic infarct (n = 128)	Non-splenic infarct (n = 377)	p value
Age	61.9 (15.9)	61.7 (15.9)	0.170
Male	74 (57.8)	216 (57.3)	0.918
**Triage level**			0.956
Level I	3 (2.34)	6 (1.59)	
Level II	39 (30.5)	116 (30.7)	
Level III	81 (63.3)	241 (63.9)	
Level IV	5 (3.91)	14 (3.71)	
**Initial vital signs**			
Fever (BT > 38 °C)	13 (10.2)	10 (2.65)	0.006
Tachycardia (HR > 100)	44 (34.4)	64 (17.0)	< 0.001
Tachypnea (RR > 20)	14 (10.9)	25 (6.63)	0.106
Hypotension (SBP < 90)	3 (2.34)	8 (2.13)	0.491
Desaturation (SpO_2_ < 90%)	1 (1.59)	2 (1.79)	0.766
Conscious change (GCS < 14)	4 (3.31)	6 (1.59)	0.361
qSOFA ≥ 1	28 (21.9)	46 (12.2)	0.011
**Previous medical history**			
Hypertension	59 (46.1)	38 (10.1)	< 0.001
Diabetes mellitus	30 (23.4)	160 (42.4)	< 0.001
Chronic liver disease	13 (10.2)	175 (46.4)	< 0.001
COPD	2 (1.56)	130 (34.5)	< 0.001
Chronic kidney disease	15 (11.7)	124 (32.9)	< 0.001
Malignancy	12 (9.38)	109 (28.9)	< 0.001
Atrial fibrillation	30 (23.4)	31 (8.22)	< 0.001
Congestive heart failure	15 (11.7)	96 (25.5)	< 0.001
**Laboratory abnormality**			
Leukocytosis (WBC > 10,000)	76 (65.0)	114 (30.2)	< 0.001
Thrombocytopenia (platelet < 100 K)	28 (23.9)	26 (6.97)	< 0.001
CRP elevation (> 100)	19 (23.5)	17 (8.50)	0.001
BUN elevation (> 30)	6 (8.11)	28 (18.4)	0.104
ALT elevation (> 120)	2 (2.20)	10 (4.17)	0.672
**Prognosis**			
Length of stay	14.3 (12.7)	3.2 (8.48)	< 0.001
ICU admission	18 (14.1)	6 (1.59)	< 0.001
Mortality	8 (6.25)	0 (0)	< 0.001

### The cases at risk of deterioration

**Figure 1:**
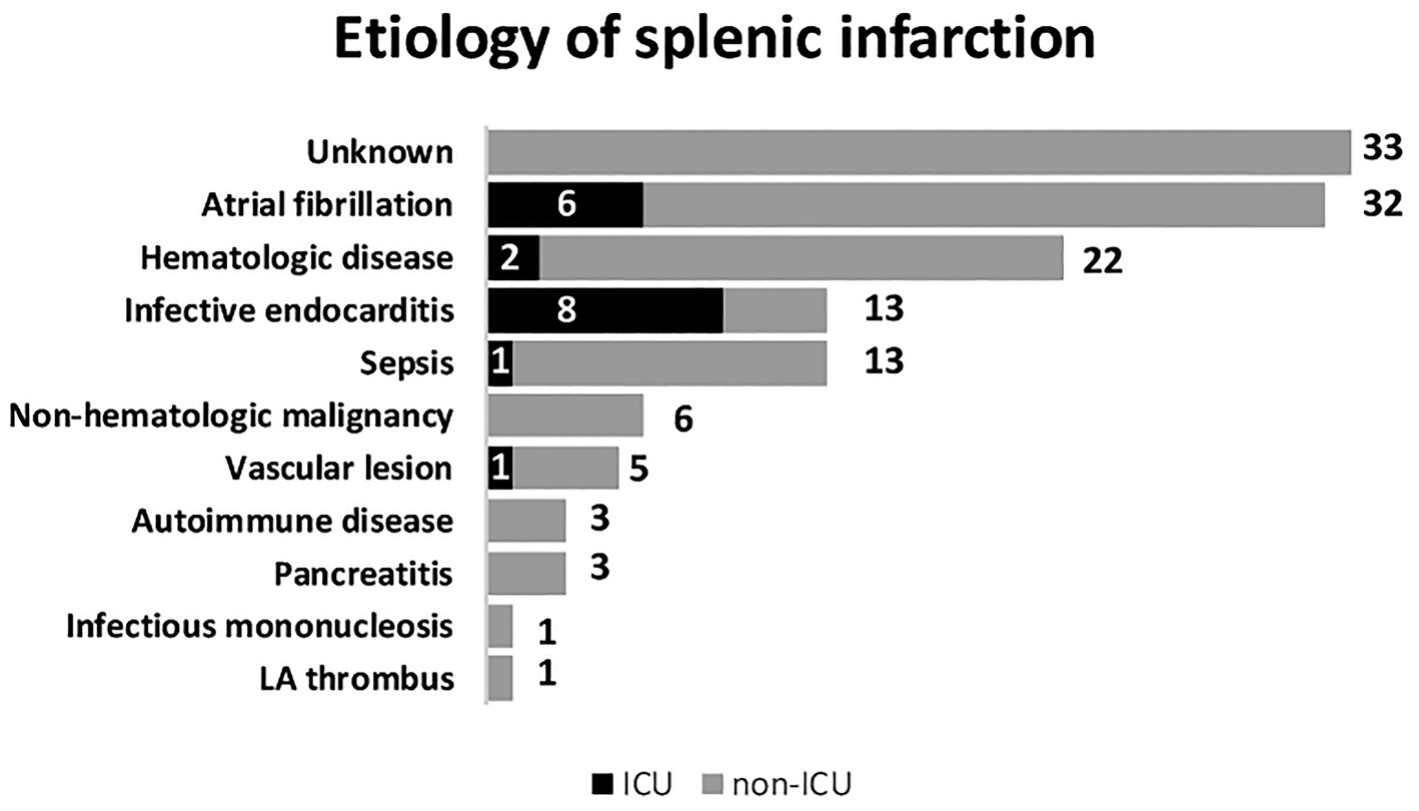
Illustration of the summary of case outcomes, stratified by confirmed etiology.

In investigating the predicting factor for ICU admission among splenic infarction patients, the univariate logistic analysis yielded five variables with a p-value of less than 0.1: tachypnea (p = 0.019), a past history of diabetes mellitus (p = 0.09), a past history of atrial fibrillation (p = 0.09), a positive qSOFA score (p = 0.026), and CRP elevation (p = 0.011). In the multivariate analysis, only tachypnea (OR = 5.18) and CRP elevation (OR = 4.95) are still statistically significant (Table 4). For the predicting factor of mortality, the variables with a p-value of less than 0.1 included fever (p = 0.0003), tachycardia (p = 0.0501), tachypnea (p = 0.0377), a positive qSOFA score (p = 0.0049), a cancer history (p = 0.0281), thrombocytopenia (p = 0.0145), and CRP elevation (p = 0.0033). In multivariate analysis, only fever (OR = 18.1), a positive qSOFA score (OR = 8.9), and a past history of malignancy (OR = 7.48) remained in the final model (Table 4). A detailed list of patients admitted to the ICU and their chief complaint, past medical history, final confirmed etiology, and prognosis are provided in Table 5. Figure 1 illustrates the summary of case outcomes stratified by confirmed etiology. A total of 33 cases were unable to classify into specific underlying etiology and are thought to be idiopathic. As can be seen, a higher percentage of ICU admission was observed in splenic infarction cases caused by infective endocarditis (61.5%) and atrial fibrillation (18.8%).

**Table 4:** Analysis of predictive factors for ICU admission and mortality using multivariate logistic regressions with stepwise selection. The threashold for inclusion were variables with a significance level of less than 0.1 in univariate analysis.

	OR (95% CI)	p value
**ICU admission**		
CRP > 100	5.18 (1.40–19.2)	0.014
Respiratory rate > 20/min	4.95 (1.18–20.9)	0.029
**Mortality**		
Fever (BT > 38 °C)	18.1 (3.06–107)	0.001
qSOFA ≥ 1	8.95 (1.58–50.6)	0.013
Malignancy history	7.48 (1.02–55.1)	0.048

**Table 5:** Patient list of ICU admission.

Case#	Age	Gender	Chief complaint	Past medical history	Etiology	Operation	Death
1	73	M	LUQ pain	HTN	Infective endocarditis	N	Y
2	41	M	Fever	CHF, Thromboembolism history	Infective endocarditis	Y	N
3	80	M	Epigastric pain	HTN	Infective endocarditis	N	N
4	57	M	Epigastric pain	Af, HTN, thromboembolism history	Atrial fibrillation	N	N
5	47	M	LUQ pain	None	Splenic artery occlusion	N	N
6	83	F	LUQ pain	Af, CAD, old CVA	Atrial fibrillation	N	N
7	76	M	LUQ pain	Af, HTN, CAD	Infective endocarditis	Y	N
8	73	F	Left flank pain	Af	Atrial fibrillation	N	N
9	66	M	Fever and weakness	HTN, old CVA	Lymphoma	N	Y
10	38	F	Fever	None	Infective endocarditis	N	N
11	26	M	Fever	HIV, syphilis	Cryptococcol meningitis	N	Y
12	54	F	Left abdominal pain	Myelodysplastic syndrome, DM, CKD, liver cirrhosis	Infective endocarditis	Y	Y
13	78	F	LUQ pain	HTN	Chronic myeloid leukemia	Y	N
14	68	M	Left abdominal pain	Af, HTN, CHF	Atrial fibrillation	N	N
15	47	M	LUQ pain	Af	Rheumatic heart disease	N	N
16	51	M	Left abdominal pain	None	Infective endocarditis	Y	N
17	64	M	Fever	None	Infective endocarditis	Y	N
18	69	F	Conscious change	HTN	Infective endocarditis	N	Y

## Discussion

This study included EMR data from multiple centers and utilized a case–control matched design to highlight the risks and prognostic factors of splenic infarction. Of all patients presenting to the emergency department with left upper quadrant pain, we provide plausible clues for differentiating splenic infarction from other diseases in the clinical environment. We also documented the morbidity and mortality of splenic infarction and associated clinical conditions in the emergency department. This study is the largest ED-based retrospective cohort study of splenic infarction and included patients from multiple medical centers. This is also the only one match control study that compared splenic infarction patients with others who presented with similar conditions.

Despite the variability of presenting symptoms, abdominal pain remains the most common symptom of splenic infarction, followed by tachycardia and nausea. Similar to previous studies^7,8^, half of our patients complained about left upper quadrant pain. Some of the studies reported a lower proportion of abdominal pain complaints that may be very possibly due to the sampling method, such as a radiology database-inclusion study design^3^. Some splenic patients may present with very mild symptoms and be first managed in an outpatient setting. In these cases, incidental findings of splenic infarction can be detected due to lowered threshold for abdominal CT scanning. When facing undifferentiated patients with similar symptoms, past medical history should also be taken into consideration. Although there were a broad variety of comorbidities in our control group because of the tertiary medical center setting, the evidence indicates a distinct difference in terms of past histories between the control and the case cohorts. Specifically, the case group possessed a much higher proportion of hypertension and atrial fibrillation patients.

Literatures reported that elevation of WBC and LDH were common in splenic infarction^8,19^. Our data revealed similar results that over half of patients (64.7%) had leukocytosis. Although few patients were tested for LDH, almost all had elevated values (9/11). For image workups, with the infusion of intravenous contrast medium, splenic infarction can be readily seen on CT scans with single or multiple well-defined regions of reduced attenuation^12^. The outline of this field is often wedge-shaped or irregular. It can also reveal the location and extent of the infarct area and other target organ lesions. High accuracy of CT examination offers effective treatment in the acute setting. Ultrasound is another diagnostic tool. A broad variety of appearances can be seen, including single or multiple, rounded or wedge-shaped, echo-free, hypoechoic, and hyperechoic lesions^20^. However, the infarcted area may not be detected in the acute phase because different echogenicity is established after one day between infarcted and normal tissue^5^. Although splenic infarction tends to resolve without invasive therapy, ultrasound can be crucial in short-term follow-up for early recognition of possible complications such as pseudocysts, abscesses, hemorrhage, or rupture^20^.

Our in-hospital mortality rate was 6.92%. A high mortality rate of up to 34% was reported by Frippiat et al.^19^. Since splenic infarction typically has a benign course, the prognosis depends largely on predisposing etiology. Like previous studies, the main cause of mortality or morbidity was the severe predisposing disease rather than splenic infarction. Valve replacement or repair surgery was performed in 38.4% of the patients (5/13) with infective endocarditis, which was consistent with one previous publication (25–50%)^21^. The two main causes for splenic infarction patients to be admitted to the ICU were cardioembolisms induced by atrial fibrillation and infectious endocarditis. Various embolic events can be present in patients with infective endocarditis though they may be clinically silent. Previous studies reported a proportion ranging from 10 to 20% of splenic infarctions in patients with endocarditis^22,23^. This crucial predisposing disease brings us to attention since that high ICU admission (9/13, 69.2%) and mortality rate(4/13, 30.7%) are found in infective endocarditis accompanying splenic infarction. We also found that a higher level of CRP, tachypnea, a positive qSOFA score, and fever are either associated with ICU admission or death. In these patients, infective endocarditis or severe inflammatory disorder were usually the most important etiology causing splenic infarction. The above prognostic features may indicate a more serious condition while others can be treated conservatively.

Thromboembolism is the leading underlying predisposing etiology of splenic infarction in the current study. In addition to atrial fibrillation, many other conditions could be related to thromboembolism. Hematologic disease, for instance, constitutes a significant part (21/130, 16.2%) of splenic infarction. Previous studies reported a wider range percentage of hematologic disease from 10 to 59%^7^. Ku et al., in their recent study, reported a 2-year cumulative incidence of venous thromboembolism of 5.2% and 4.5% among AML and ALL, respectively^24^. Another meta-analysis revealed that the global incidence rates of venous or arterial events were 5.3% and 1.1%, respectively, among all lymphoma patients^25^. In the current study, 12 of our patients had myeloproliferative neoplasms comparable with the literature^26^. The overall risk of thrombosis is 1–3% per patient-year in patients with essential thrombocythemia^27^ or primary myelofibrosis^28^. We should therefore raise a high index of suspicion for thrombotic risks in patients with known myeloproliferative neoplasms.

For non-hematologic malignancy, we found two patients with hepatocellular carcinoma, two with cholangiocarcinoma, and two with pancreatic adenocarcinoma. Splenic infarction in hepatocellular carcinoma was formerly reported in sorafenib use^29^, post transcatheter arterial chemoembolization^30^, and post ethanol ablation^31^. Two recent case reports revealed splenic infarction in cholangiocarcinoma, depicted as Trousseau's syndrome^32,33^. There appears to be a high association with the hyper-coagulation system induced by mucin-producing adenocarcinoma. In particular, Cox et al. reported a high correlation of splenic infarction in patients with pancreatic cancer^6^, possibly owing to the short distance between the pancreas and the splenic vessels. For autoimmune diseases, three of our patients had systemic lupus erythematosus (SLE), all diagnosed unexpectedly. The pathogenesis of thrombosis in SLE consists of hypercoagulability, premature atherosclerosis, and vasculitis. The risk of thrombosis remains high even in the absence of aPL^34^. In a 10-year multicenter study of 1000 patients with SLE from 7 European countries, 92 patients had thrombosis(9.2%), which was the second frequent cause of death^35^.

## Limitations

Our study had several limitations. First, this is a single-country study. The race and ethnicity in Taiwan are relatively homogenous. To generalize the results of this study, clinicians must consider the differences in cardioembolic incidences across races. Second, due to the retrospective nature, this study is inherently limited by the missing data. For a control group patient, who presented to the ED with LUQ pain, and whose tentative diagnosis from the physician is urolithiasis or pleuric pain, the laboratory examination arranged would be very different from that of a splenic infarction patient. However, it is also difficult to conduct a prospective comparative study among splenic infarction patients due to its scarcity and sporadic nature.

## Conclusion

In this retrospective matched case–control study, splenic infarction patients often presented with left upper abdominal pain and tachycardia. A history of hypertension or atrial fibrillation or a laboratory result of leukocytosis or thrombocytopenia may provide clues for clinicians to include splenic infarction in the differential list. Among the patients diagnosed with splenic infarction, those with an underlying etiology of infectious endocarditis may be prone to deterioration or ICU admission. Since leukocytosis is common in splenic infarction patients, an additional test of CRP or other biomarkers directly related to bacterial infection may be of prognostic value.
